# Fall of viral and bacterial pneumonia hospitalizations following COVID-19 pandemic mitigation strategies: a central Italian Region retrospective study

**DOI:** 10.1007/s11739-023-03213-y

**Published:** 2023-02-07

**Authors:** Irma Clemente, Silvano Junior Santini, Pierpaolo Vittorini, Enrico Pallotta, Gaia Sinatti, Antonella Fontana, Leondino Mammarella, Ermenegildo Rancitelli, Clara Balsano

**Affiliations:** 1 Department of Life, Health and Environmental Sciences-MESVA, School of Emergency-Urgency Medicine, University of L’Aquila, 67100 L’Aquila, Italy; 2 Francesco Balsano Foundation, Via Giovanni Battista Martini 6, 00198 Rome, Italy; 3 U.O.S.D. Servizio Gestione Flussi Informativi e Statistica Sanitaria, L’Aquila, Italy; 4 Unità Operativa Complessa Controllo di Gestione, Pescara, Italy

**Keywords:** Community-acquired pneumonia, Bacterial pneumonia, Viral pneumonia, Containing measures, PPE, COVID-19, SHR

## Abstract

Community-Acquired Pneumonia (CAP) represents one of the first causes of hospitalization and death in the elderly all over the world and weighs heavily on public health system. Since the beginning of the COVID-19 (CoronaVirus Disease-19) pandemic, everybody’s behavior was forced to change, as the result of a global lockdown strategy and the obligation of using personal protection equipment (PPE). We aimed to evaluate how the mitigation strategies adopted to fight SARS-CoV-2 (Severe Acute Respiratory Coronavirus Syndrome 2) infection have influenced hospitalizations due to CAP in two different Local Health Boards (LHBs) of central Italy. We considered two main periods of observation: before and after the national start of lockdown, in two Abruzzo’s LHBs. We analyzed 19,558 hospital discharge records of bacterial and viral CAP. Excluding SARS-CoV2 infection, a significant decrease in CAP hospitalizations was observed. Through the analysis of Diagnosis Related Group (DRG) values, we highlighted a significant saving of founds for the Regional Health Service. The enactment of social distancing measures to contain COVID-19 spread, brought down admissions for bacterial and viral pneumonia. Our study emphasizes that costs for hospitalizations due to CAP could be drastically reduced by mask wearing and social distancing.

**Supplementary Information:**

The online version contains supplementary material available at 10.1007/s11739-023-03213-y.

## Introduction

Community-acquired pneumonia (CAP) is an important cause of mortality and morbidity worldwide [1]. According to WHO, lower respiratory infections were the fourth leading cause of death in 2019: 2.6 million people died, accounting 3.3% of overall mortality [2, 3]. The incidence of pneumonia increases with age and is often associated with several comorbidities, like cardiovascular (CV) and lung diseases, diabetes mellitus (DM) and malignancies, that play a crucial role in management of CAP [4].

CAP represents one of the major causes of hospitalization. Its incidence is estimated between 5 and 11 cases for 1,000 inhabitants in Europe [5]. CAP transmission occurs mainly by sneezing and coughing, however, CAP diffusion might be more complex, depending on host features, environment, interpersonal interactions, and comorbidities [6]. Interestingly, recent studies highlighted the fundamental role of intra-human interactions and asymptomatic carriers in bacterial CAP development, especially in S. Pneumoniae-related pneumonia [7–13].

In the last decades, it was estimated that hospitalizations for CAP have increased about 34%, with a 30-days mortality of 19.8% in adults with more than 60 years [14, 15].

With an aging population that continues to grow, in the next future, our health care system will face multi-comorbidities patients, the rising of hospitalization lengths and multiple resources consumption; thus, the number of older patients hospitalized for pneumonia will significantly increase as well as the associated healthcare costs [16–18]. In Europe, the annual cost is around €10 billion, whereas in the United States the economic burden related with pneumonia is estimated to be more than $17 billion annually [19, 20].

The guidelines for empirical treatment of CAP contemplate the use of antibiotics, from a single macrolide-based therapy to a combined Beta-lactam/macrolide or to fluoroquinolones therapy in non-complicated situations. However, it should be considered that resistant organisms frequently do not respond to the usual first-line treatment, thus increasing significantly indirect costs [21].

Besides that, respiratory viruses also greatly contribute to pneumonia in both adults and children and, when methodically tested for, are more frequently detected than bacteria [22]. During the last 2 years, a new cause of viral pneumonia was identified in Wuhan, China. It was sustained by SARS-CoV-2, a virus that causes interstitial and bilateral pneumonia, leading from asymptomatic to mild-severe conditions, even requiring intensive care treatment. In March 2020, the Italian government, following the indications of the WHO (World Health Organisation), introduced drastic measures of confinement and social distancing to limit SARS-CoV-2 spreading.

Among the various rules, the most impacting behaviors on people's daily life were social distancing, frequent hand disinfection and the use of personal protective equipment (PPE) as face masks [23, 24]. Depending on the hospital structures capacity, each Italian Region imposed restrictive measures, starting from the mandatory use of PPEs and up to global lockdown [25].

The aim of our study was to evaluate, during the first wave of pandemic, in two different areas of Abruzzo Region, the effect of the introduction of the restrictive measures on hospitalization rates for viral or bacterial CAP.

## Materials and methods

We conducted a retrospective observational study on 19,558 cases, analyzing patient’s HDR (Hospital Discharge Records) from February 1^st^ 2019 to March 31^st^, 2021; these patients were admitted to LHB1 and LHB3 hospitals in Abruzzo Region. We analyzed age-adjusted Standardized Hospitalization Rate (SHR) per 10,000 inhabitants of bacterial and viral non-COVID CAP. To better pursuit our aim in understanding the effects caused by SARS-CoV-2 pandemic spreading, we observed CAP diagnosis in two different main sub-periods: 1) the pre-pandemic period from February 2019 to February 2020; 2) the pandemic period from March 2020 to March 2021. Specifically, we observed SARS-CoV-2 pneumonia from the beginning of its spreading, from January 2020 to March 2021. The specific ICD-9 codes used to analyze HDR are the following: 481, 482 and subgroups, 483 and subgroups for bacterial pneumonia; 485, 486, 487, 480 and subgroups for viral pneumonia; 07,889, 4848, 04,311, 04,312, 48,041, 48,042, 51,891, 51,892 for SARS-CoV-2 infection with various manifestations; 466, 490, 491, 492, 494, 496, 516.3, 518.82, 519.8, 770.0 used to identify other pneumopathies.

### Characteristics of the two LHBs

We compared LHB1 (Avezzano-Sulmona-L’Aquila) to LHB3 (Pescara) patient’s SHR, because they represent the population of two very different areas of Abruzzo Region, with different susceptibility to CAP.

The territory of Avezzano-Sulmona-L’Aquila is 100% mountain area, while the province of Pescara is almost hilly with coastal areas. The territorial factor has implications on climate changes, going from a Mediterranean climate along the coasts to a continental one as soon as one proceeds toward the inland areas, where the Gran Sasso massif Apennine mountains stands. L’Aquila (LHB1 chief town) in fact is located 720 m above sea level and is one of the coldest cities in Italy, while Pescara stands along the sea.

The distribution of population is slightly different in the two areas. According to the latest report from ISTAT, a total of 292,356 people live in the province of L’Aquila, 314,689 people live in the province of Pescara. The average age is 46.4 y.o. (years old) in the province of L’Aquila, 45.6 y.o. in the province of Pescara, with old-age-dependency index of 39.8 y.o. and 37.9 y.o., respectively. The main differences are in the orographic characteristics. The surface of LHB1, with an extension of 5,034 Km^2^, covers the 47% of the entire region territory. The dispersion of the population in the LHB1 territory takes particular importance, expressed by an average density of 59 inhabitants/Km^2^. Otherwise, the surface of LHB3, with an extension of 1,023 Km^2^, represents only 11% of Abruzzo surface even though it hosts 24% of the population of Abruzzo with a density of 259 inhabitants/Km^2^. Average annual temperature (C°) is 9 ± 7 in LHB1 and 15 ± 7 in LHB3, with annual rainfall, respectively, of 81 ± 21 and 63 ± 16 mm [26].

### Data collection integration and quality

The analyzed dataset is made up of the HDR collected by LHB1 and LHB3, anonymised for privacy. The datasets from the two LHBs were non-homogeneous (i.e., they used different column names, encoding and semantics), thus required a preliminary pre-processing consisting of: 1) data-mapping to solve the schematic heterogeneity, and 2) data-conflicts resolutions (especially with reference to dates). All HDR were filtered by all ICD9-CM codes reported in material and methods section.

### Statistical methods

We analyzed the data with both descriptive and inferential statistics. Standard deviations, absolute and relative frequencies were used for descriptive statistics [27].

We also calculated the age-adjusted monthly hospitalization rates per 10,000 inhabitants (SHRs), using the direct method, that considers the different population sizes and age distributions, to enable a comparison between non-homogeneous areas (LHB1 and LHB3) and homogeneous periods (both pre-pandemic and post-pandemic periods lasted 1 year). For the inferential statistics, we calculated the confidence intervals for the age-adjusted hospitalization rates. To this aim, we used the method of Fay and Feuer [28], based on the gamma distribution, to avoid calculating negative numbers for the lower confidence limit, that may come back with the normal approximation. We plotted the monthly SHRs, for the different LHBs, marking the pre/post-pandemic phases, for a direct understandability of our findings. Finally, non-overlapping 95% confidence intervals indicated a statistically significant difference.

All analyses were performed using the R 4.0.4 software, with the “epitools” package to calculate the age-adjusted hospitalization rates [29, 30].

## Results

Of the 19,558 total HDR, 11,875 belong to LHB1 and 7,683 to LHB3; 11,388 were recorded during the pre-pandemic phase and 8,170 during the pandemic one. Table 1 shows the demographic differences in hospitalizations for bacterial and viral pneumonia between the two LHBs in the period before and after the entry into force of the containment regulations for the virus.

**Table 1:** Demographic differences in bacterial and viral pneumonia (excluded SARS-CoV-2) hospitalizations across LHBs in the pre-pandemic and pandemic period

Demographics	LHB1			LHB3		
	All	PRE	Pandemic	All	PRE	Pandemic
Total hospitalizatio*n* (%)	11,875	7421 (62.5)	4454 (37.5)	7683	3967 (51.6)	3716 (48.4)
Bacterial pneumonia						
Total pneumonia (%)	2207	1770 (80.2)	437 (19.8)	856	595 (69.5)	261 (30.5)
Male (%)	1264	1029 (58.1)	235 (53.8)	537	367 (61.7)	170 (65.1)
Female (%)	943	741 (41.9)	202(46.2)	319	228 (38.3)	91 (34.8)
< 65 (%)	566	480 (84,8)	86 (15,2)	287	226 (78,7)	61 (21,3)
≥ 65 (%)	1641	1290 (78,6)	351 (21,4)	569	369 (64,8)	200 (35,2)
Viral pneumonia						
Total pneumonia (%)	4297	3163 (73.6)	1134 (16.4)	3849	2582 (67.1)	1267 (32.9)
Male (%)	2282	1682 (53.2)	600 (52.9)	2165	1455 (56.4)	710 (56.0)
Female (%)	2015	1481 (42.8)	534 (47.1)	1684	1127 (43.6)	557 (44.0)
< 65 (%)	1008	813 (80,6)	195 (19,4)	1017	730 (71,7)	287 (28,3)
≥ 65 (%)	3289	2350 (71,4)	939 (28,6)	2832	1852 (65,9)	980 (34,1)

### Effect of restrictive measures on CAP-related SHR

In the analyzed pandemic period (March 2020–March 2021), the number of hospitalizations for bacterial CAP and viral CAP (excluding SARS-CoV-2 diagnosis) was significantly lower than in the pre- pandemic period (February 2019-February 2020). Prevalence of bacterial CAP hospitalization reduced from 1770 to 437 in LHB1, and from 595 to 261 in LHB3 (Table 1). Viral CAP hospitalization decreased from 3163 to 1134 in LHB1 and from 2582 to 1267 in LHB3 (Table 1).

For bacterial pneumonia, in the pre-pandemic phase, SHR for LHB1 was 48.9 (CI_95%_ [46.5–51.3]), whereas it was 16.2 (CI_95%_ [14.9–17.7]) for LHB3 (Table 2). The difference was statistically significant. After the introduction of the anti-contagion rules in both LHBs, the SHR for bacterial pneumonia significantly decreased with a hospitalization rate that remained lower in LHB3 than in LHB1 (SHR for LHB1 was 11.4 (CI_95%_ [10.3–12.6]) and it was 6.6 (CI_95%_ [5.8–7.5]) for LHB3). This difference was also significant (Table 2).

**Table 2:** SHR differences in bacterial and viral (excluded SARS-CoV-2) pneumonia hospitalizations across LHBs in the pre-pandemic and pandemic period (statistically significant differences are in bold: * vs pre-pandemic of its LHB, # vs LHB1)

	LHB1	LHB3
	adj.rate	adj.rate
Bacterial pneumonia SHR		
PRE	48.9 (CI_95%_ [46.5–51.3])	**16.2**^**#**^ (CI_95%_ [14.9–17.7])
Pandemic	11.4* (CI_95%_ [10.3–12.6])	6.6*^,#^ (CI_95%_ [5.8–7.5])
Viral pneumonia SHR		
PRE	87.0 (CI_95%_ [83.8–90.2])	**68.0**^**#**^ (CI_95%_ [65.2–70.8])
Pandemic	28.7* (CI_95%_ [27.0–30.3])	32.3*^,#^ (CI_95%_ [30.4–34.2])

Regarding viral pneumonia, during the pre-pandemic phase, SHR for LHB1 was 87.0 (CI_95%_ [83.8–90.2]), whereas for LHB3 was 68.0 (CI_95%_ [65.2–70.8]). The difference was statistically significant. Otherwise in the pandemic phase SHR was 28.7 (CI_95%_ [27.0–30.4]) for LHB1, and 32.3 (CI_95%_ [30.4–34.2]) for LHB3. This difference was statistically significant, with a SHR for LHB3 surprisingly higher than LHB1 (Table 2).

**Fig. 1:**
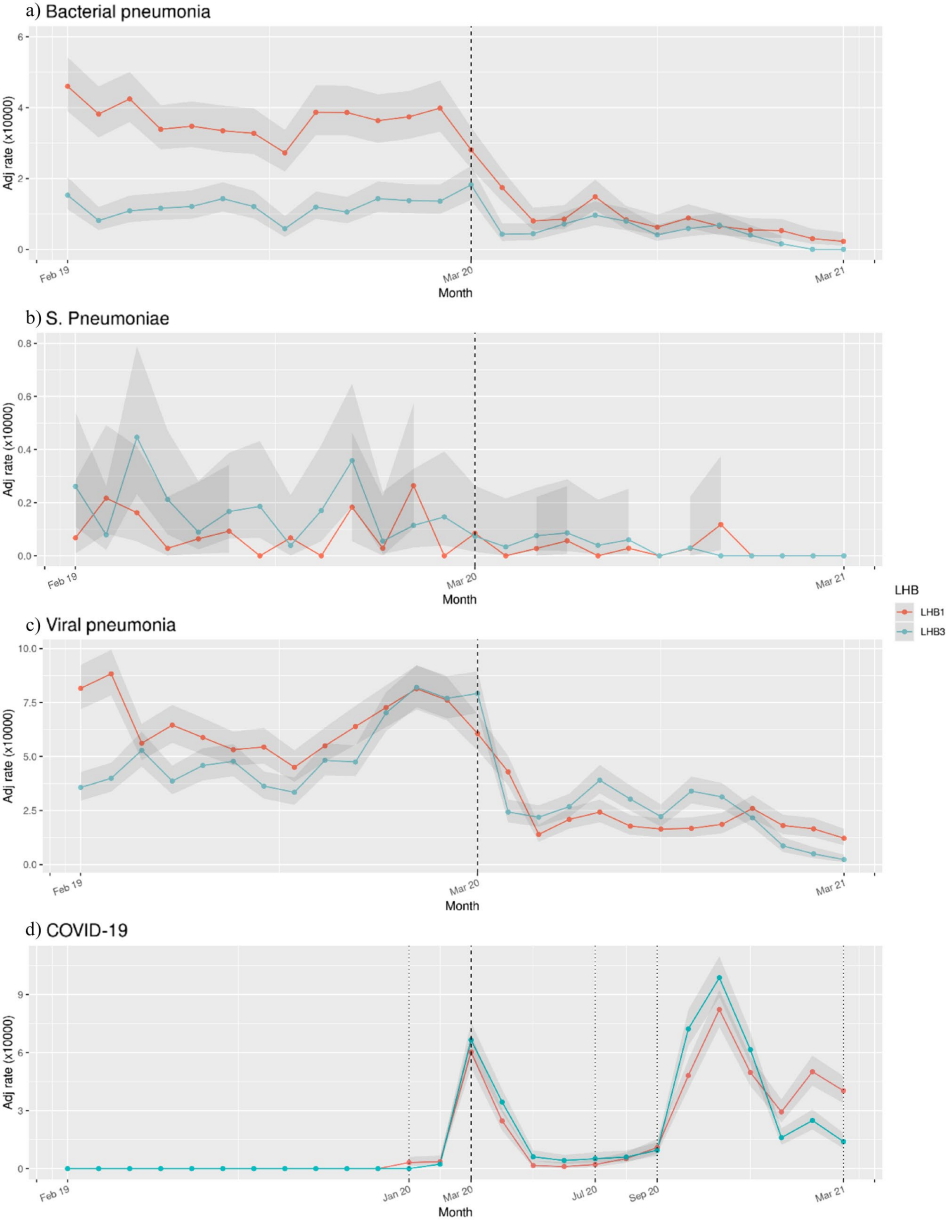
Temporal changes in age adjusted SHR for bacterial pneumonia (**a**), pneumococcal pneumonia (**b**), viral pneumonia (excluded SARS-CoV-2) (**c**) and SARS-CoV-2 infection (**d**) in LHB1 and LHB3 during pre-pandemic period (February 2019–February 2020) and pandemic period (March 2020–March 2021)

Figure 1 gives more details about the SHR, by reporting its value with confidence intervals for all analyzed months, divided for viral, bacterial, and SARS-CoV-2 pneumonia. Worth noting, during the pandemic phase, the SHR for pneumonia remained almost stable, whereas the SHR for SARS-CoV-2 peaks in the second wave.

**Fig. 2:**
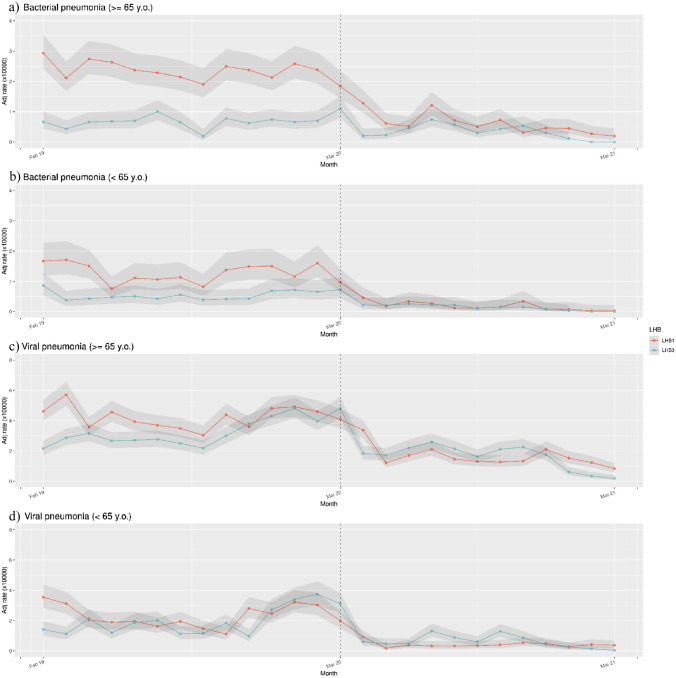
Temporal changes in SHR for bacterial pneumonia in people ≥ 65 y.o. (**a**) and under 65 y.o. (**b**) and for viral pneumonia (excluded SARS-CoV-2) in people ≥ 65 y.o. (**c**) and under 65 y.o. (**d**) in LHB1 and LHB3 during the pre-pandemic period (February 2019–February 2020) and pandemic period (March 2020–March 2021)

Figure 2 shows the monthly SHR for bacterial and viral pneumonia stratified by age (≥ or < 65 y.o.) in the two LHBs.

Focusing on bacterial pneumonia in elderly people (≥ 65 y.o.), during the pre-pandemic phase, SHR was higher in LHB1 (31.5 CI_95%_ [29.8–33.4]) than LHB3 (8.7 (CI_95%_ [7.9–9.8])), the difference was statistically significant. In the pandemic period, LHB1’s SHR dropped to 8.7 (CI_95%_ [7.8–9.6]), whereas LHB3’s SHR dropped to 4.7 (CI_95%_ [4.1–5.5]), the difference was statistically significant. Even younger population (< 65 y.o.), during the pre-pandemic period, displayed a higher SHR in LHB1 than in LHB3 (17.3, CI_95%_ [15.8–19.0] vs 7.5, CI_95%_ [6.5–8.5]); anti-contagion norms decreased hospitalization rates in both LHBs without showing statistically significant differences in the two areas (2.7, CI_95%_ [2.2–3.4] vs 1.9, CI_95%_ [1.4–2.4]). To investigate if age might influence the SHR results, we better analyzed the SHR data of S. Pneumoniae, demonstrating that the decrease in hospitalization was similar in patients ≥ 65 y.o. and < 65 y.o. (see supplementary Tab. S4). In the LHB3, this phenomenon is even more pronounced in patients < 65 y.o.

Regarding viral pneumonia, elderly people (≥ 65 y.o.), during the period before pandemic, showed a greater SHR in LHB1 (56.0, CI_95%_ [53.7–58.4]) than LHB3 (42.3, CI_95%_ [40.3–44.4]). During the pandemic period, SHR for LHB1 was reduced to 22.4 (CI_95%_ [20.1–23.1]) and to 22.8 (CI_95%_ [21.4–24.3]) for LHB3. This difference was not statistically significant (Fig. 2).

The SHR of younger population (< 65 y.o.), during the pre-pandemic phase, was 30.9 (CI_95%_ [28.8–33.2]) for LHB1 and 25.7 (CI_95%_ [23.8–27.6]) for LHB3. The difference was statistically significant. After the application of measures to contain virus spreading, SHRs for viral pneumonias dropped in both LHBs, but SHR values remained significantly higher in LHB3 respect to LHB1 (9.4, CI_95%_ [8.4–10.6] vs 6.2, CI_95%_ [5.4–7.2], respectively).

Tables S2 and S3 report the SHR for the main comorbidities related to CAP hospitalizations. It should be noted that SHR for all comorbidities was significantly lower in LHB3 compared to LHB1.

### Decrease in expenditure of CAP-related hospitalization

Finally, we estimated the healthcare expenditure and its related National Health Service refund for CAP hospitalization by DRG (Diagnosis Related Group) tables, considering the period before and after 9th of March 2020. Each diagnosis of CAP, either bacterial or viral, was multiplied for its respective DRG value.

A total of 51,238,736 Euro was recognized as refund for bacterial and viral hospitalizations in the period from 1st of February 2019 to 31st of March 2021 in the two LHBs, respectively, 16,438,112 Euro (32.1%) for bacterial CAP stays and 34,800,624 Euro (67.9%) for viral ones. Mean cost for each hospitalization was 5,366 Euro for bacterial and 4,272 Euro for viral CAP stays. In the pre-pandemic phase, the 71.4% (36,572,568 Euro) of this amount was spent for CAP admissions, while the remaining 28.6% (14,666,168 Euro) was spent in the pandemic phase, till 31st of March 2021.

DRG for bacterial CAP dropped from 8,960,266 to 2,153,156 Euro in LHB 1, with a decrease of the 76% of the costs, and from 3,989,741 to 1,334,949 (decreased of 66.5%) Euro in LHB3. DRG for viral CAPs decreased from 12,478,072 to 5,199,090 (decreased of 58.4%) Euro in LHB 1 and from 11,144,489 to 5,978,973 (46.4% decrease) Euro for LHB 3.

## Discussion

The first wave of pandemic has forced governments and local administrations to introduce extraordinary health measures to counteract the spread of SARS-CoV-2.

This study was conducted to evaluate the effect of PPE and restrictive measures on SHR of both bacterial and viral CAP (excluding SARS-CoV-2 CAP). We analyzed two periods: the pre-pandemic from February 2019 to February 2020, and the pandemic from March 2020 to March 2021.

The study highlights a significant decrease in SHR for bacterial and viral CAP, in both areas, after the introduction of social distancing and PPE, resulting in a constant decrease in the hospitalization curve for CAP (Fig. 1 and Table 2). We observed a significative SHR difference between the two LHBs, in both the periods of study; this difference was mainly noticed among bacterial CAP (Fig. 1). In line with literature findings, bacterial and viral CAP prevalence was higher in males (Table 1) [31].

In the pandemic period, mortality rate for viral and bacterial CAP significantly decreased in both areas, however LHB3 showed a lower death rate respect to LHB1 (Table S2 and S3). The discrepancy might be explained by the higher prevalence of comorbidities in LHB1, in particular COPDs SHR in LHB1 area were five folds higher than in LHB3 (Table S2 and S3).

Finally, our study analyzed the healthcare expenditure by bacterial and viral CAP DRG analysis, before and after the social-restrictive decree of the 9th of March 2020. DRG calculation represents the value of money refund by the Italian Health Service to Regional Health Service. The economic expenditure was significantly reduced during the two observed periods: DRGs decreased of 40,1% ranging from 36,572,568 to 14,666,168 Euro. In particular, DRG related to bacterial CAP decrease by 76% in LHB1 and by 66.5% in LHB3, whereas viral CAP by 58.4% in LHB1 and by 46.4% in LHB3.

Our results are in line with Drug Italian Agency 2020 Report which claims a decrease in the consumption of antibiotics compared to 2019. This change could be largely due to the prevention measures implemented during the pandemic. Accordingly with our data, the precautions taken, such as the use of masks and social distancing, have been reported to be effective in reducing the consumption of antibiotics in 2020: 18.2% in Italy and to 23.9% in Abruzzo [32]. Also Ryabak et al. analysis demonstrates CAP reduction, especially in children [33]

This study has some limitations. We analyzed only hospitalized CAP, but it should be considered that pandemic had a great impact on sanitary system organization, limiting access to Emergency departments due to both the overcharging of sanitary transport system and the fear of contagion. Despite this, the database of Italian Institute of Statistics (ISTAT) reports that, in Abruzzo Region, in the first semester of 2020, deaths related to community acquired pneumonia were comparable to those of the same period of the previous 5 years [34].

The COVID-19 pandemic has given us an important lesson: social distancing measures should be assumed not only in specific environments (e.g., hospitals, nursing homes, medical clinics, etc.), but adopting these measures could be also useful in cold season to protect the categories of frail people from the infection of endemic microorganisms. Many works worldwide confirmed how mitigation strategies implemented the fight against virus spreading and are an efficient measure [35, 36]. Undoubtedly, the enforcement of primary and secondary prevention represents the only viable and realistic long-term strategy for COVID-19 pandemic mitigation [37]. We know that is very utopian and unrealistic to expect that the individual citizen could be invited to wear permanent mask, but we encourage the scientific society to evaluate the possibility of recommending the “social mask wearing” as a hygienic rule especially in some seasons or in crowded places.

## Supplementary Information

Below is the link to the electronic supplementary material.

Supplementary file1 (DOCX 23 KB)

## Data Availability

The datasets used and/or analyzed during the current study are available from the corresponding author on reasonable request.
