# Supplementary material for: Fall of viral and bacterial pneumonia hospitalizations following COVID-19 pandemic mitigation strategies: a central Italian Region retrospective study
Source: Intern Emerg Med. 2023 Feb 7;18(4):1181–9. doi: 10.1007/s11739-023-03213-y (PMC9904871; doi:10.1007/s11739-023-03213-y)
Supplement: Supplementary file 1 — Supplementary file1 (DOCX 23 KB) [file 11739_2023_3213_MOESM1_ESM.docx]

**Table S1**. Demographic differences hospitalizations for Pneumococcal pneumonia between the LHBs in the pre-pandemic and pandemic period.

| **Demographics** | **LHB1** | | | **LHB3** | | |
| --- | --- | --- | --- | --- | --- | --- |
|  | **All** | **PRE** | **Pandemic** | **All** | **PRE** | **Pandemic** |
| Total hospitalization (%) | 11875 | 7421 (62.5) | 4454 (37.5) | 7683 | 3967 (51.6) | 3716 (48.4) |

| **PNEUMOCOCCAL PNEUMONIA** | | | | | | |
| --- | --- | --- | --- | --- | --- | --- |
| Total pneumonia (%) | 47 | 35 (74,5) | 12 (25,5) | 94 | 78 (83) | 16 (17) |
| Male (%) | 21 | 17 (80,9) | 4 (19,1) | 53 | 42 (79,2) | 11 (20,8) |
| Female (%) | 26 | 18 (69,2) | 8 (30,8) | 41 | 36 (87,8) | 5 (12,2) |
| <65 (%) | 20 | 16 (80) | 4 (20) | 37 | 35 (94,6) | 2 (5,4) |
| ≥65 (%) | 27 | 19 (70,3) | 8 (29,7) | 57 | 43 (75,4) | 14 (24,6) |

**Table S2**. Death and comorbidities rates differences in bacterial pneumonia between the LHBs in the pre-pandemic and pandemic period (statistically significant differences are in bold: * vs pre-pandemic of its LHB, # vs LHB1).

|  | **Deaths** | |
| --- | --- | --- |
|  | **LHB1** | **LHB3** |
|  | adj.rate | adj.rate |
| PRE | 37.4 (CI_95%_ [35.3, 39.5]) | **19.2^#^** (CI_95%_ [17.7, 20.8]) |
| Pandemic | **15.5*** (CI_95%_ [14.2, 16.9]) | **10.7*^,#^** (CI_95%_ [9.6, 11.9]) |
|  | **Main Comorbidity** | |
|  | **Hypertension** | |
|  | **LHB1** | **LHB3** |
|  | adj.rate | adj.rate |
| PRE | 33.7 (CI_95%_ [31.8, 35.8]) | **5.4^#^** (CI_95%_ [4.7, 6.3]) |
| Pandemic | **10.1*** (CI_95%_ [9.0, 11.2]) | **3.2*^,#^** (CI_95%_ [2.6, 3.9]) |
|  | **Heart disease** | |
|  | **LHB1** | **LHB3** |
|  | adj.rate | adj.rate |
| PRE | 44.5 (CI_95%_ [42.2, 46.8]) | **6.9^#^** (CI_95%_ [6.0, 7.8]) |
| Pandemic | **12.6^#^** (CI_95%_ [11.5, 13.9]) | **5.9*** (CI_95%_ [5.1, 6.8]) |
|  | **Diabetes** | |
|  | **LHB1** | **LHB3** |
|  | adj.rate | adj.rate |
| PRE | 38.0 (CI_95%_ [35.8, 40.2]) | **5.0^#^** (CI_95%_ [4.3, 5.8]) |
| Pandemic | **9.6*** (CI_95%_ [8.5, 10.7]) | **1.8*^,#^** (CI_95%_ [1.3, 2.3]) |
|  | **Chronic Obstructive Pulmonary Disease** | |
|  | **LHB1** | **LHB3** |
|  | adj.rate | adj.rate |
| PRE | 35.0 (CI_95%_ [33.0, 37.1]) | **6.6^#^** (CI_95%_ [5.7, 7.6]) |
| Pandemic | **12.7*** (CI_95%_ [11.5, 14.0]) | **2.3*^,#^** (CI_95%_ [1.8, 2.9]) |

**Table S3**. Death and comorbidities rates differences in viral pneumonia between the LHBs in the pre-pandemic and pandemic period (statistically significant differences are in bold: * vs pre-pandemic of its LHB, # vs LHB1).

|  | **Deaths** | |
| --- | --- | --- |
|  | **LHB1** | **LHB3** |
|  | adj.rate | adj.rate |
| PRE | 34.7 (CI_95%_ [32.7, 36.8]) | 32.9 (CI_95%_ [31.0, 34.9]) |
| Pandemic | **20.52*** (CI_95%_ [19.0, 22.2]) | **17.4*^,#^** (CI_95%_ [16.0, 18.9]) |
|  | **Main Comorbidity** | |
|  | **Hypertension** | |
|  | **LHB1** | **LHB3** |
|  | adj.rate | adj.rate |
| PRE | 22.8(CI_95%_ [2212, 24.5]) | 24.5 (CI_95%_ [22.8, 26.3]) |
| Pandemic | **15.5*** (CI_95%_ [14.2, 17.0]) | **12.3*^,#^** (CI_95%_ [11.1, 13.6]) |
|  | **Heart disease** | |
|  | **LHB1** | **LHB3** |
|  | adj.rate | adj.rate |
| PRE | 46.6(CI_95%_ [44.4, 49.0]) | **24.5^#^** (CI_95%_ [4.3, 5.8]) |
| Pandemic | **24.4*** (CI_95%_ [22.7, 26.1]) | **10.5*^,#^** (CI_95%_ [9.4, 11.7]) |
|  | **Diabetes** | |
|  | **LHB1** | **LHB3** |
|  | adj.rate | adj.rate |
| PRE | 20.5(CI_95%_ [18.9, 22.0]) | **12.5^#^** (CI_95%_ [11.3, 13.7]) |
| Pandemic | **8.5*** (CI_95%_ [7.6, 9.6]) | **5.6*^,#^** (CI_95%_ [4.9, 6.5]) |
|  | **COPD Chronic Obstructive Pulmonary Disease** | |
|  | **LHB1** | **LHB3** |
|  | adj.rate | adj.rate |
| PRE | **18.1** (CI_95%_ [16.7, 19.7]) | **14.7^#^** (CI_95%_ [4.3, 5.8]) |
| Pandemic | **6.8*** (CI_95%_ [5.9, 7.7]) | **5.4*** (CI_95%_ [4.6, 6.3]) |

**Table S4**. SHR differences in pneumococcal pneumonia between the LHBs in the pre-pandemic and pandemic period (statistically significant differences are in bold: * vs pre-pandemic of its LHB, # vs LHB1).

|  | **S. Pneumococcal pneumonia SHR** | |
| --- | --- | --- |
|  | **LHB1** | **LHB3** |
|  | adj.rate | adj.rate |
| PRE | 1.17 (CI95% [0.8, 1.7]) | 2.32^#^ (CI95% [1.8, 2.9]) |
| Pandemic | 0.34* (CI95% [0.17, 0.65]) | 0.39* (CI95% [0.22, 0.68]) |
|  | **S. Pneumococcal pneumonia SHR <65 yo** | |
|  | adj.rate | adj.rate |
| PRE | 0.64 (CI_95%_ [0.36, 1.06]) | 1.26 (CI_95%_ [0.87, 1.77]) |
| Pandemic | 0.15 (CI_95%_ [0.04, 0.42]) | 0.06* (CI_95%_ [0.01, 0.25]) |
|  | **S. Pneumococcal pneumonia SHR <65 yo** | |
|  | adj.rate | adj.rate |
| PRE | 0.53 (CI_95%_ [0.32, 0.87]) | 1.06 (CI95% [0.76, 1.46]) |
| Pandemic | 0.19 (CI_95%_ [0.08, 0.43]) | 0.33* (CI_95%_ [0.18, 0.61]) |
